# Supplementary material for: Causal Relationship between Aging and Anorexia Nervosa: A White-Matter-Microstructure-Mediated Mendelian Randomization Analysis
Source: Biomedicines. 2024 Aug 16;12(8):1874. doi: 10.3390/biomedicines12081874 (PMC11351342; doi:10.3390/biomedicines12081874)
Supplement: Supplementary file 1 [file biomedicines-12-01874-s001.zip › Supplementary Figure/Figure S4/Singe SNP plot of MR analysis for TL to ubm-b-1970.pdf]

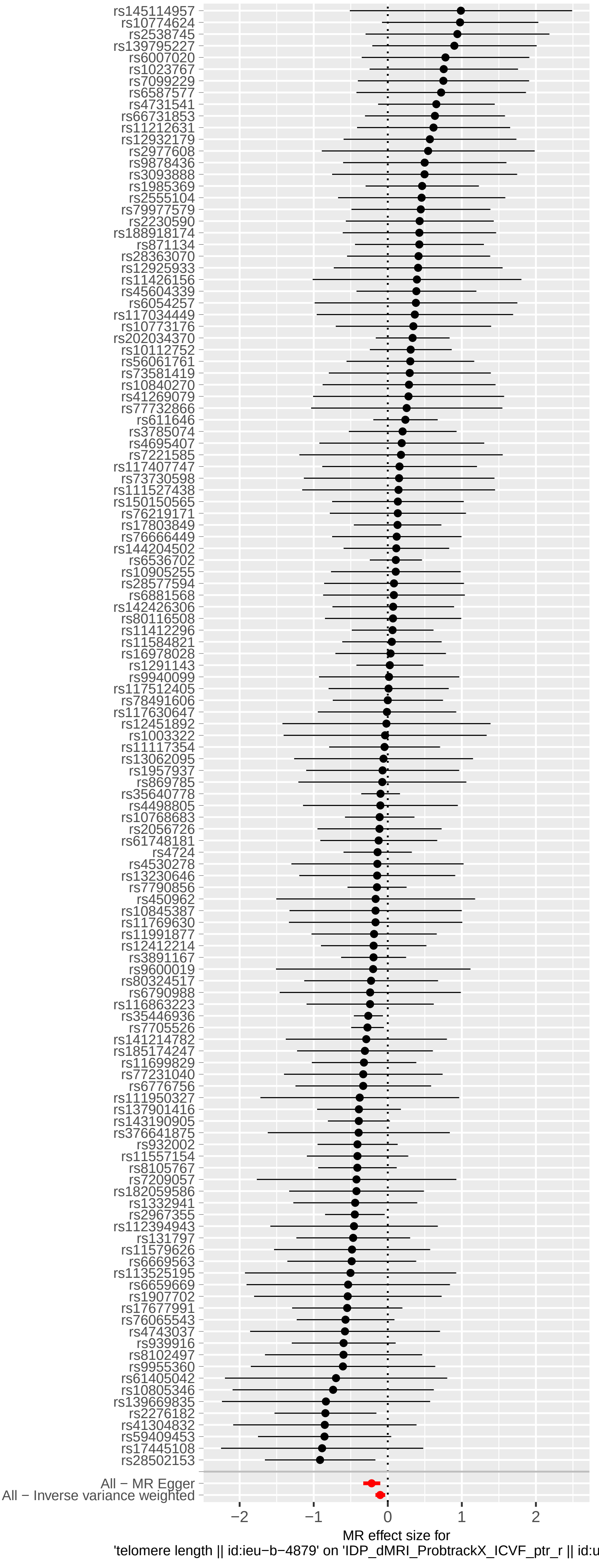
rs145114957
rs10774624
rs2538745
rs139795227
rs6007020
rs1023767
rs7099229
rs6587577
rs4731541
rs66731853
rs11212631
rs12932179
rs2977608
rs9878436
rs3093888
rs1985369
rs2555104
rs79977579
rs2230590
rs188918174
rs871134
rs28363070
rs12925933
rs11426156
rs45604339
rs6054257
rs117034449
rs10773176
rs202034370
rs10112752
rs56061761
rs73581419
rs10840270
rs41269079
rs77732866
rs611646
rs3785074
rs4695407
rs7221585
rs117407747
rs73730598
rs111527438
rs150150565
rs76219171
rs17803849
rs76666449
rs144204502
rs6536702
rs10905255
rs28577594
rs6881568
rs142426306
rs80116508
rs11412296
rs11584821
rs16978028
rs1291143
rs9940099
rs117512405
rs78491606
rs117630647
rs12451892
rs1003322
rs11117354
rs13062095
rs1957937
rs869785
rs35640778
rs4498805
rs10768683
rs2056726
rs61748181
rs4724
rs4530278
rs13230646
rs7790856
rs450962
rs10845387
rs11769630
rs11991877
rs12412214
rs3891167
rs9600019
rs80324517
rs6790988
rs116863223
rs35446936
rs7705526
rs141214782
rs185174247
rs11699829
rs77231040
rs6776756
rs111950327
rs137901416
rs143190905
rs376641875
rs932002
rs11557154
rs8105767
rs7209057
rs182059586
rs1332941
rs2967355
rs112394943
rs131797
rs11579626
rs6669563
rs113525195
rs6659669
rs1907702
rs17677991
rs76065543
rs4743037
rs939916
rs8102497
rs9955360
rs61405042
rs10805346
rs139669835
rs2276182
rs41304832
rs59409453
rs17445108
rs28502153
All − MR Egger
All − Inverse variance weighted
−2
−1
0
1
2
MR effect size for
'telomere length || id:ieu−b−4879' on 'IDP_dMRI_ProbtrackX_ICVF_ptr_r || id:u
